# Multivariate Analysis of Preoperative Magnetic Resonance Imaging Reveals Transcriptomic Classification of *de novo* Glioblastoma Patients

**DOI:** 10.3389/fncom.2019.00081

**Published:** 2019-12-12

**Authors:** Saima Rathore, Hamed Akbari, Spyridon Bakas, Jared M. Pisapia, Gaurav Shukla, Jeffrey D. Rudie, Xiao Da, Ramana V. Davuluri, Nadia Dahmane, Donald M. O'Rourke, Christos Davatzikos

**Affiliations:** ^1^Center for Biomedical Image Computing and Analytics, Perelman School of Medicine, University of Pennsylvania, Philadelphia, PA, United States; ^2^Department of Radiology, Perelman School of Medicine, University of Pennsylvania, Philadelphia, PA, United States; ^3^Department of Pathology and Laboratory Medicine, Perelman School of Medicine, University of Pennsylvania, Philadelphia, PA, United States; ^4^Division of Neurosurgery, Children Hospital of Philadelphia, Philadelphia, PA, United States; ^5^Christiana Care Health System, Philadelphia, PA, United States; ^6^Brigham and Women's Hospital, Boston, MA, United States; ^7^Department of Biomedical Informatics, Northwestern University Feinberg School of Medicine, Chicago, IL, United States; ^8^Department of Neurological Surgery, Weill Cornell Medicine, New York, NY, United States; ^9^Department of Neurosurgery, Perelman School of Medicine, University of Pennsylvania, Philadelphia, PA, United States

**Keywords:** transcriptomic classification, glioblastoma, multivariate analysis, brain tumors classification, biomarkers

## Abstract

Glioblastoma, the most frequent primary malignant brain neoplasm, is genetically diverse and classified into four transcriptomic subtypes, i. e., classical, mesenchymal, proneural, and neural. Currently, detection of transcriptomic subtype is based on *ex vivo* analysis of tissue that does not capture the spatial tumor heterogeneity. In view of accumulative evidence of *in vivo* imaging signatures summarizing molecular features of cancer, this study seeks robust non-invasive radiographic markers of transcriptomic classification of glioblastoma, based solely on routine clinically-acquired imaging sequences. A pre-operative retrospective cohort of 112 pathology-proven *de novo* glioblastoma patients, having multi-parametric MRI (T1, T1-Gd, T2, T2-FLAIR), collected from the Hospital of the University of Pennsylvania were included. Following tumor segmentation into distinct radiographic sub-regions, diverse imaging features were extracted and support vector machines were employed to multivariately integrate these features and derive an imaging signature of transcriptomic subtype. Extracted features included intensity distributions, volume, morphology, statistics, tumors' anatomical location, and texture descriptors for each tumor sub-region. The derived signature was evaluated against the transcriptomic subtype of surgically-resected tissue specimens, using a 5-fold cross-validation method and a receiver-operating-characteristics analysis. The proposed model was 71% accurate in distinguishing among the four transcriptomic subtypes. The accuracy (sensitivity/specificity) for distinguishing each subtype (classical, mesenchymal, proneural, neural) from the rest was equal to 88.4% (71.4/92.3), 75.9% (83.9/72.8), 82.1% (73.1/84.9), and 75.9% (79.4/74.4), respectively. The findings were also replicated in The Cancer Genomic Atlas glioblastoma dataset. The obtained imaging signature for the classical subtype was dominated by associations with features related to edge sharpness, whereas for the mesenchymal subtype had more pronounced presence of higher T2 and T2-FLAIR signal in edema, and higher volume of enhancing tumor and edema. The proneural and neural subtypes were characterized by the lower T1-Gd signal in enhancing tumor and higher T2-FLAIR signal in edema, respectively. Our results indicate that quantitative multivariate analysis of features extracted from clinically-acquired MRI may provide a radiographic biomarker of the transcriptomic profile of glioblastoma. Importantly our findings can be influential in surgical decision-making, treatment planning, and assessment of inoperable tumors.

## Introduction

Glioblastoma is the most frequent primary malignant brain tumor with grim prognosis, despite aggressive combination of therapies (Stupp et al., 2017), and is characterized by inter- and intra-patient heterogeneity at radiographic, histologic, and molecular fronts, thereby providing opportunities for sub-classification, prognostication, and adoption of targeted therapeutic approaches (Aum et al., 2014; Lemée et al., 2015).

There is mounting evidence that different glioblastoma patients show different levels of sensitivity to therapeutic approaches depending on their distinct genetic characterization. It has been suggested earlier that glioblastoma should not be considered a single disease, but rather should be categorized into four transcriptomic subtypes, i.e., classical, mesenchymal, proneural, and neural (Verhaak et al., 2010). These subtypes present very distinct molecular biomarkers such as collective loss in chromosome 10 and amplification of chromosome 7 in classical subtype, largest occurrence of focal hemizygous deletions of a region at 17q11.2, encompassing *NF1 gene*, in mesenchymal subtype, aberrations in PDGFRA and mutations in *IDH1* in proneural subtype, and presence of *GABRA1, SYT1, NEFL*, and *SLC12A5* in neural subtype (Verhaak et al., 2010). In a recent study by Park et al. it has been shown that subtype-specific genetic aberrations have potential to serve as predictive markers and therapeutic targets (Park et al., 2019).

The determination of the molecular profile of the tumors leads to personalized diagnosis and treatment, as different treatment options may be considered depending on the characteristics of each subtype (Phillips et al., 2006; Verhaak et al., 2010; Bhat et al., 2011). Up until now, the assessment of transcriptomic subtypes was done via molecular profiling of surgical or biopsy tissue. However, such assessment has inherent limitations of: (i) tissue sampling error that sometimes leads to missing the tumor mutation, and (ii) inability to acquire multiple specimens over the course of the disease due to invasiveness of the tissue collection procedure, thereby leading to the failure in determining molecular subtype of the tumor over the course of the treatment.

Analysis of multi-parametric magnetic resonance imaging (mpMRI) data via advanced pattern analytics methods has been progressively shown to provide rich classifications of glioblastoma and its surrounding brain tissue, and has helped identifying relationships between MRI biomarkers and transcriptomic subtypes in gliomas (Gutman et al., 2013; Naeini et al., 2013; Gevaert et al., 2014; Pisapia et al., 2015; Macyszyn et al., 2016; Khened et al., 2019). For instance, the proneural subtype has shown lower levels of contrast enhancement; the mesenchymal subtype has presented lower levels of non-enhanced tumors and intensity in peritumoral edema region (Gutman et al., 2013); the classical subtype has associated necrosis and sharped edges of the edema region (Gevaert et al., 2014). A model to predict the mesenchymal subtype was also proposed (Naeini et al., 2013).

However, this classification scheme has been difficult to translate into clinical practice due to several complicating factors. First, existing literature has found associations between imaging features and individual subtypes (Naeini et al., 2013). Second, most studies to date have used basic imaging sequences only or have used very few hand-crafted imaging features, failing to leverage the power of computationally extracting and selecting imaging features (recently called radiomics), and analyzing them through advanced pattern analysis methods to build a more powerful predictive model (Gutman et al., 2013; Gevaert et al., 2014; Macyszyn et al., 2016). As literature increasingly acknowledges the tumor spatial and temporal heterogeneity, there is a parallel focus on extracting extensive features of the tumor and its surrounding peritumoral region toward providing a better characterization of patients. Furthermore, analysis of advanced mpMRI data can provide more details, which might not be available in conventional imaging.

This study aims to determine the transcriptomic subtypes of *de novo* glioblastoma patients by multivariately assessing imaging features from routine clinically-acquired scans, reflecting tumor biological properties such as angiogenesis, proliferation, cellularity, and peritumoral infiltration. Identifying these transcriptomic subtypes may allow enrollment of patients into targeted clinical trials, longitudinal profiling of the tumor, and assessment of treatment response.

## Materials and Methods

### Study Setting and Data Source

This study evaluates a group of 112 primary glioblastoma patients, diagnosed between 2006 and 2013 at the Hospital of the University of Pennsylvania (HUP), having pre-operative mpMRI (T1, T2, T1-Gd, T2-FLAIR). A subset of these patients (*n* = 89) had additional diffusion tensor imaging (DTI) and dynamic susceptibility contrast-enhanced (DSC) MRI imaging available. The proposed classification models were developed on *n* = 112 patients using conventional imaging only (T1, T2, T1-Gd, T2-FLAIR), whereas the subset of the patients (*n* = 89) was used to further analyze the imaging properties of different subtypes. The overall analysis was carried out on HUP dataset, and the findings were then replicated independently in The Cancer Genomic Atlas glioblastoma (TCGA-GBM) dataset (Clark et al., 2013; Scarpace et al., 2013) (*n* = 60), part of the International Brain Tumor Segmentation (BraTS) challenge dataset (Menze et al., 2015; Bakas et al., 2018), having the same set of pre-operative mpMRI. Expert manual segmentations for this dataset were downloaded from The Cancer Imaging Archive (TCIA) website (Bakas et al., 2017a,b). The study population was uniformly distributed and did not have any statistically significant difference based on clinical and demographical factors. All experiments were approved by the Institutional Review Board (IRB) of the University of Pennsylvania (approval no: 706564) and written informed consent was obtained from all patients. All experiments were carried out in accordance with the guidelines and regulations of the approved IRB.

### Transcriptomic Subtyping

After pathologic confirmation of glioblastoma diagnosis, all tumors underwent subtyping into one of the four transcriptomic subtypes (classical = 21, mesenchymal = 31, proneural = 26, neural = 34). For this subtyping, we used an isoform-level assay classifier initially constructed using exon array data from glioblastoma samples in TCIA (Verhaak et al., 2010). It was then translated into a clinically applicable platform, where expression of desired transcripts was measured using reverse transcriptase–quantitative polymerase chain reaction (RT-qPCR) (Pal et al., 2014). RNA was isolated from the tissue samples using Tri Reagent (Sigma). A high-capacity complementary DNA reverse transcriptase kit (Applied Biosystems) was used to reverse-transcribe the RNA, and qPCR was then performed to designate the subtype. The assay was based on the expression of 121 transcripts with four housekeeping genes as controls.

### Pre-processing Applied on the Dataset

All MRI of each patient were pre-processed using a series of image processing steps, including: (i) smoothing (i.e., reducing high frequency noise variations while preserving underlying anatomical structures) using Smallest Univalue Segment Assimilating Nucleus (SUSAN) denoising (Smith et al., 1997); (ii) correction for magnetic field inhomogeneity using N3 bias correction (Tustison et al., 2010); (iii) co-registration of all MRIs of each patient at 12-degrees of freedom for examining anatomically aligned signals at the voxel level using affine registration through the Linear Image Registration Tool (Jenkinson and Smith, 2001); (iv) skull stripping using the Brain Extraction Tool (Smith, 2002); and (v) matching of intensity profiles (histogram matching) of all MRIs of all patients to the corresponding MRIs of a reference patient.

**Figure 1:**
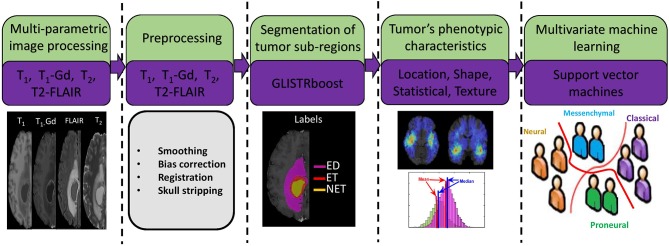
Image post-processing workflow. I. Preoperative imaging sequences (examples: T1-Gd, T2-FLAIR, T1, T2). II. Preprocessing. III. Segmentation of various subregions of tumor such as peritumoral edema region (ED), non-enhancing tumor core (NET), and enhancing tumor (ET). IV. Extraction of radiomic features in the segmented regions (ED, ET, NET) using all the imaging sequences. V. Multivariate machine learning model of support vector machines with 5-fold cross validation and sequential feature selection.

Following the pre-processing, all tumors were segmented in distinct radiographic sub-regions of peritumoral edema region (ED), enhancing tumor (ET), and non-enhancing tumor (NET) (Figure 1) using a computational algorithm [namely GLISTRboost (Gooya et al., 2011; Bakas et al., 2016)]. The segmentations were assessed by two expert readers (H.A., G.S.) and revised before image analysis, when necessary. The segmentations were transformed into a standard atlas space to produce a standardized statistical distribution atlas for quantifying the tumor spatial location.

### Radiophenotypic Tumor Characterization

The radiophenotypic characteristics of each tumor were quantified using a comprehensive and diverse set of imaging features, extracted from all tumor sub-regions (i.e., ED, ET, NET) and all MRI sequences using the Cancer Imaging Phenomics Toolkit (CaPTk) (Davatzikos et al., 2018). The feature set extracted to build the predictive model for this study comprised of (i) volumetric measurements, (ii) morphology parameters, (iii) location information, and (iv) statistical moments of the intensity distributions. The volumetric, location, and intensity statistics were calculated in 3D. The volumetric measurements include volume and surface area measurements of ED, NET, ET, tumor core (TC), which is the union of NET and ET, and whole tumor (WT), which is the combination of TC with ED. In addition, ratios of the volumes of the various tumor sub-regions and their union over the brain volume, were also calculated.

To capture the spatial distribution of each tumor, eight spatial distribution atlases were constructed as introduced in Akbari et al. (2018), two for each molecular subtype, i.e., P^(+)^ and P^(−)^ for proneural and non-proneural tumors, respectively. These distribution atlases were generated by superimposing the TC (ET+NET) segmentation labels of all patients according to their transcriptomic subtype status, i.e., superimposing the TC labels of proneural and non-proneural tumors. The similarity of the distribution pattern of an unseen tumor is then calculated by considering the intersection area between the tumor and the spatial map (Figure S1). Maximum and average frequency for each spatial distribution atlas in the intersected area are estimated, and four discrete relative values (L1, L2, L3, and L4) are used to evaluate any new unseen patient, for each subtype, thereby leading to a total of 16 location features.

$$\begin{array}{l} L_{1}=mean\left[P^{+}\right]-mean\left[P^{-}\right],\\ L_{2}=\max\left[P^{+}\right]-max\left[P^{-}\;\right],\\ L_{3}=\frac{mean\left[P^{+}\right]}{mean\left[P^{-}\;\right]},\\ L_{4}=\frac{\max\left[P^{+}\right]}{\max\left[P^{-}\right]} \end{array}$$

Moreover, the distance of various tumor sub-regions, e.g., ED, TC, from the ventricles, and the proportions of TC in each lobe of the brain have also been utilized as additional location features. The proportion of TC in various brain regions, including temporal, frontal, parietal, occipital, basal ganglia, cc fornix, insula, cerebellum, and brain stem, was calculated by mapping each image to an atlas template via a deformable registration method (Gooya et al., 2011) that not only accounts for mass effect but also takes care of inter-individual anatomical variations (Kwon et al., 2014).

Furthermore, we used first-order statistical moments of intensity distributions to quantify the phenotypic characteristics of each tumor sub-region, along with second-order statistics that describe textural properties in tumor sub-regions. A gray-level co-occurrence matrix was calculated by considering the voxels within a radius of 1 and in the 13 main directions, and texture features of contrast, correlation, energy, and homogeneity were extracted. The intensity profiles of various sub-regions of tumor were also quantified using histograms. These histograms are reflective of the changes caused by the tumor both at functional and anatomical levels, which in turn change the corresponding imaging signals, and have shown strong association with various outcome of interest (Macyszyn et al., 2016; Rathore et al., 2018). Here, each intensity distribution is divided in to 5 bins and percentage of voxels in each bin are calculated.

Morphology parameters, comprising area, perimeter, extent, solidity, and length of major- and minor-axis, were extracted from one 2D slice per tumor. In order to pick the 2D slice for extraction of morphological features, we traversed the image in the axial direction and found the slice that had largest area of tumor core.

### Feature Selection and Predictive Model Development

Support Vector Machines (SVM) (Chang and Lin, 2011), that has been extensively used in the past in medical image classification/segmentation (Lao et al., 2008; Haller et al., 2013), was used for predictive modeling in this study. We dealt the problem of classification as 4 one-vs. -rest classification problems. We trained a separate SVM to discriminate between one transriptomic subtype and the rest of the subtypes, such as classical vs. others, mesenchymal vs. others, neural vs. others, and proneural vs. others. To confirm the robustness of the method and to ensure that estimates of accuracy would be likely to generalize to new patients, we evaluated all classifiers through 5-fold cross-validation. In each iteration of the cross-validation, feature selection and classifier's parameters optimization was performed on the training folds and the resulting classification model, developed solely on the training folds, was applied on new/unseen test fold. Sequential forward feature selection was employed at each iteration until convergence, i.e., there was no improvement over a specific threshold. The final classification performance was obtained by combining the predictions of individual classifiers. For each classifier, the particular subtype was considered positive class and the rest of the subtypes were considered negative class. The distance of the sample from the hyperplane was noted for each classifier and highest distance was chosen as the final label of the sample. For example, if proneural, neural, mesenchymal, and classical have 0.45, 3.54, −2.43, and 5.32, then the classical label was assigned to the sample.

The classification performance of the proposed models was evaluated in terms of accuracy, balanced accuracy, sensitivity, and specificity. Sensitivity and specificity refer to the percentage of correctly classified samples of positive and negative classes, respectively. Balanced accuracy is the average of the proportion corrects of each class individually, whereas accuracy is the total proportion corrects of the population.

### Statistical Analysis

The statistical analysis was performed with R (version 3.3.2, http://www.R-project.org), SPSS (version 25.0.0.0, IBM), and MatLab (version R2014b, Mathworks). For evaluation of statistically significant imaging features associated with each subtype, we used Kruskal-Wallis test (Chan et al., 1997).

## Results

### Performance of the Transcriptomic Subtype Prediction Model

The cross-validated accuracy and balanced accuracy [BA] of the obtained classifiers for classical, mesenchymal, proneural, and neural subtypes was 88.4% [BA: 81.9%], 75.9% [BA: 78.4%], 82.1% [BA: 78.9%], and 75.9% [BA: 76.9%], respectively. The overall 4-way classification among the four transcriptomic subtypes was 71% under 5-fold cross-validation experiment. Performance of the proposed prediction model is given in Table 1 where the first four columns show the result for binary classification, wherein each transcriptomic subtype is classified against the rest of the subtypes, and the last column shows the final 4-way classification accuracy obtained by combining the predictions of individual classifiers.

**Table 1:** Performance of the proposed transcriptomic subtype prediction model in terms of various performance measures.

**Classification performance**	**Imaging subtypes** ***(n)***				
	**Proneural (*n* = 26)**	**Neural (*n* = 34)**	**Mesenchymal (*n* = 31)**	**Classical (*n* = 21)**	**Overall (*n* = 112)**
Accuracy	82.14	75.89	75.89	88.39	71.00
Balanced accuracy	78.98	76.89	78.36	81.87	79.01
Sensitivity	73.08	79.41	83.87	71.43	77.68
Specificity	84.88	74.36	72.84	92.31	79.75
AUC	0.82	0.78	0.81	0.84	---

*First four rows show the result for binary classification wherein each subtype is classified against the rest of subtypes. The last row shows the final 4-way classification accuracy obtained by combining the predictions of individual classifiers*.

**Figure 2:**
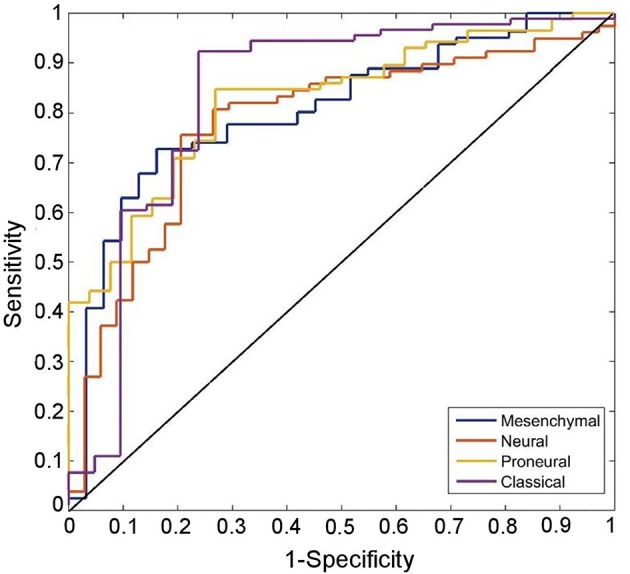
ROC curves of the predicted transriptomic subtypes are compared with chance (the diagonal line). ROC curves correctly classify proneural, neural, mesenchymal, and classical subtypes with 82.1% (sensitivity: 73.1, specificity = 84.9), 75.9% (sensitivity: 79.4, specificity = 74.4), 75.9% (sensitivity: 83.9, specificity = 72.8), and 88.4% (sensitivity: 71.4, specificity = 92.3) classification success rate, respectively.

Receiver-operating-characteristic (ROC) analysis on the given dataset yielded an area-under the-curve (AUC) of 0.82, 0.78, 0.81, and 0.84, for proneural, neural, mesenchymal, and classical subtypes, respectively (Figure 2).

### Important Phenotypic Characteristics of Different Transcriptomic Subtypes

**Figure 3:**
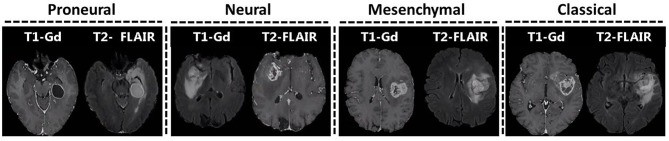
Representative images of different subtypes.

Along with evaluating the predictive performance of the model, we assessed individual features with the most predictive value. Our results have shown that specific subtypes have quite distinct quantitative imaging features, which can be utilized (Table 2, Figure 3). The main characteristics of the obtained imaging signature show that the mesenchymal subtype (in comparison with other subtypes) have lower T2 and T2-FLAIR signal in peritumoral edematous/invaded region, ET of lower eccentricity, NET of higher eccentricity, and higher volumes of ET, ED and WT. The proneural subtype, compared with the other subtypes, included signals of lower and uniform T1-Gd in ET. The neural subtype showed signals of higher T2-FLAIR in ED and lower eccentricity of NET, and the classical subtype showed smaller surface area of ED and WT.

**Table 2:** Important imaging characteristics that distinguish each subtype from the rest of the subtypes.

**Imaging subtypes** ***(n)***			
**Proneural (*n* = 26)**	**Neural (*n* = 34)**	**Mesenchymal (*n* = 31)**	**Classical (*n* = 21)**
Lower Signal in ET (T1-Gd)	Higher signal in ED (T2-FLAIR)	Lower signal in ED (T2-FLAIR)	Surface area (ED, WT)
Higher uniformity in ET (T1-Gd)	Lower eccentricity (NET)	Lower signal in ED (T2)	
		Lower eccentricity (ET)	
		Higher eccentricity (NET)	
		Bigger volume (ED, ET, WT)	

### Replication of the Proposed Model in TCIA Dataset

The predictive performance of the proposed model was also evaluated in an independent replication dataset of pre-operative glioblastoma patients, downloaded from TCIA (Bakas et al., 2017a), by applying the model trained on the discovery (i.e., HUP) dataset. The information about the molecular subtypes of TCIA patients was acquired from existing studies (Verhaak et al., 2010; Park et al., 2019). The four models, pertaining to four different molecular subtypes, trained on HUP dataset were applied to the patients in the replication (i.e., TCIA) cohort. The final molecular status of each patient in the replication dataset was obtained by combining the predictions of individual classifiers, as done in the discovery dataset, leading to 69% classification success rate compared to 25% chance in 4-way classification accuracy.

## Discussion

We identified an *in vivo* radiographic signature of transcriptomic subtypes in glioblastoma by using quantitative multivariate analysis of mpMRI in a non-invasive manner, and further attempted to provide patho-physiological associations of the most distinctive imaging features. An important existing study has demonstrated the potential that deep learning techniques can be used for identifying associations between brain imaging phenotypes and genomic characteristics (Khened et al., 2019). The hereby proposed method is different from existing literature (Macyszyn et al., 2016; Khened et al., 2019) on the breadth of extracted mpMRI-based features, leading to an extensive radiographic signature. The proposed signature sheds light into the anatomical and pathological characteristics of the tumor, via macroscopic imaging features summarizing tumor characteristics related to water concentration, blood-brain barrier breakage, cell density, uniformity/heterogeneity, and geometric variations. We have achieved these findings utilizing routine mpMRI scans acquired under current clinical practice for glioblastoma, without the need to utilize any molecular imaging methods. We evaluated our model via a cross-validation mechanism in the HUP dataset, and also performed a multi-institutional validation to demonstrate generalizability. Potential applications of this signature include facilitating the assessment of transcriptomic status for patients with inadequate tissue. In a recent study by Park et al., it has been shown that subtype-specific genetic aberrations have potential to serve as predictive markers and therapeutic targets (Park et al., 2019). Therefore, in case of subtype-targeted clinical trials, it becomes very important to distinguish one particular subtype from the rest. The automatic distinction of these subtypes leads to personalized diagnosis and treatment, as different options may be considered depending on the histologic characteristics of different subtypes.

### Biological Explanation of Quantitative Features of Different Subtypes

Toward gaining an understanding about the biological developments that induce different mutation status, we analyzed in isolation each individual feature that we used to develop our classification models. The analysis revealed that each subtype had an accompanying distinct and comprehensive set of radiographically relatable features (Table 2). The main findings from comparing the features of different transcriptomic subtypes, in ET, NET, and ED, are as follows:

1. Regions of lower and uniform T1-Gd signal in proneural subtype, suggestive of less blood–brain barrier compromise;
2. Areas of lower water content in mesenchymal subtype, reflected by T2-FLAIR and T2-weighted imaging, consistent with the characteristics of dense tissue;
3. Larger surface area of ED and WT in mesenchymal subtype, which points toward deep infiltration and migratory nature of the tumor;
4. Smaller surface area of ED and WT in classical subtype, supporting a radiographic phenotype of compact and less migratory nature of the tumor;
5. Major to minor axes ratios, associated with NET in neural subtype and ET/NET in mesenchymal subtype, were different from other subtypes (Table 2). The major axis was characterized by the longest possible 2D distance in a region; minor axis is vertical to the major axis. This eccentricity measure is suggestive of regular/spherical NET in neural subtype and irregular NET in mesenchymal subtype.
6. Regions of relatively lower contrast of T1 imaging sequence in ET in neural subtype, suggestive of more uniform T1 signal (Table 2).

It is important to note that despite several discriminative features, neither of these features is sufficient enough to predict transcriptomic subtype on each patient basis. However, synergistic integration of these features via appropriate machine learning yielded reasonable sensitivity and specificity in predicting subtype on an individual patient basis, thereby underscoring the potential of multivariate analysis methods.

### Discriminative Power of Advanced MRI (DTI and DSC-MRI) Modalities

Advanced MRI sequences were evaluated to probe their discriminative power, compared to that of structural (conventional) imaging, i.e., T1, T2, T2-Flair, and T1-Gd. It is worth mentioning that these imaging sequences were not utilized to develop the classification models, rather only to analyze the diffusion and perfusion characteristics of a subset of these patients. These additional sequences comprised derivatives of DTI [i.e., fractional anisotropy (FA), apparent diffusion coefficient (ADC), radial diffusivity (RAD), axial diffusivity (AX)], as well as DSC-MRI derivatives, i.e., percentage signal recovery (PSR), peak height (PH), and relative cerebral blood volume (rCBV).

**Figure 4:**
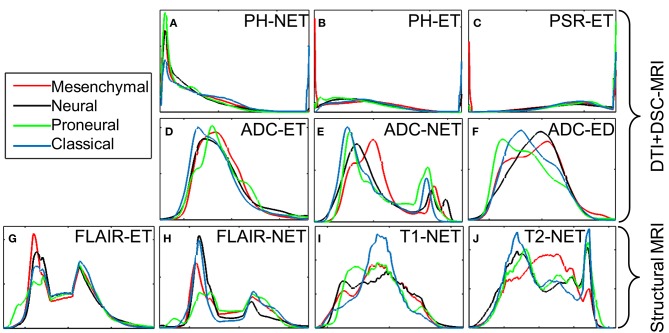
Intensity histograms display the diffusion and perfusion measures of different transcriptomic subtypes as measured by the DTI and DSC-MRI signals. **(A)** PH intensity in NET; **(B)** PH intensity in ET; **(C)** PSR intensity in ET; **(D)** ADC intensity in ET; **(E)** ADC intensity in NET; **(F)** ADC intensity in ED; **(G)** T2-Flair intensity in ET; **(H)** T2-Flair intensity in NET; **(I)** T1 intensity in NET; **(J)** T2 intensity in NET. The measures displayed in the first two rows are for analysis only; these measures have not been used for building the model.

Imaging derivatives of DTI are reflective of the water diffusion process, which is partially affected by the architecture and density of tumor cells (Lu et al., 2003), in brain. The classical subtype has larger regions of lower ADC determined by the histograms (Figure 4) in NET (*p* = 2.27 × 10^−08^) and ET (*p* = 1.97 × 10^−07^) of the tumor, suggestive of less watery, and denser tumors. Imaging derivatives of DSC-MRI enumerate microvasculature and hemodynamics characteristics of the tumor (Wintermark et al., 2005; Tykocinski et al., 2012). When volume of brain tumors exceeds a certain critical limit, the consequential ischemia activates the discharge of angiogenic factors, which in turn endorses vascular proliferation and eventually leads to the formation of leaky and torturous tumor vessels (Lev and Hochberg, 1998; McDonald and Choyke, 2003; Bullitt et al., 2005; Hicklin et al., 2005; Essock-Burns et al., 2011; Thompson et al., 2011; Swami, 2013; Jensen et al., 2014). These imaging derivatives also steered toward some key findings. The classical subtype showed imaging features in agreement with highly vascular tumor, as shown by the PH in ET (*p* = 1.54 × 10^−15^) and NET (*p* = 4.00 × 10^−06^), revealing increased and compromised micro-vascularity compared to other subtypes. On the other hand, the proneural subtype had increased PSR in ET, indicative of lower micro-vascularity compared to other subtype.

### Clinical Relevance and Impact

The assessment of transcriptomic subtype of glioblastoma via analysis of tissue specimen can be limited due to sampling error, and reluctance for longitudinal assessment of the status due to invasive nature of surgery. Our proposed imaging signature has potential to address both these limitations, since mpMRI facilitates assessment and monitoring of the tumor in its entirety in a repeatable manner. Further, the non-invasive imaging signature captures the heterogeneity of the whole tumor extent, instead of the analysis of one tissue specimen, therefore provides a global perspective of the transcriptomic status of a tumor. Our imaging signature is derivative of mpMRI that is routinely acquired for glioblastoma patients, therefore, is ready for immediate translation to the clinic. While the current method focuses on non-invasive assessment of transcriptomic subtype status, the same approach could also be used for molecular assessment in general. Further, the proposed non-invasive imaging signature can be applied to recurrent glioblastoma, with the goal of determining transcriptomic subtype status before, during, and after the treatment. This would help in non-invasive monitoring of dynamic changes in transcriptomic subtypes as response to targeted therapeutic approaches and consequently would in turn allow for tailoring the adopted therapies.

## Conclusion

We can quantify important imaging characteristics within various sub-regions of the tumor and detect its transcriptomic subtype only by examining mpMRI data using advanced analytical methods and without the need of advanced genetic testing. The present study extracts an extensive set of quantitative imaging phenomic features from structural MRI sequences, and employs these variables via machine learning techniques to non-invasively distinguish transcriptomic glioblastoma subtypes. This molecular classification, due to its distinct phenotypic pattern derived from routine MRI, renders our imaging signature of increased likelihood for effective and immediate translation into clinical practice. The use of cross-validation within HUP dataset and the replication of our findings on TCIA dataset provide confidence in the generalizability of these subtypes and the proposed method on other datasets.

## Data Availability Statement

Publicly available datasets were analyzed in this study. This data can be found here: TCGA.

## Ethics Statement

The studies involving human participants were reviewed and approved by the Institutional Review Board (IRB) of University of Pennsylvania (approval no: 706564). The patients/participants provided their written informed consent to participate in this study.

## Author Contributions

SR, HA, SB, and CD: conceptualization. SR: methodology, software, formal analysis, and writing—original draft. HA, SB, JP, GS, JR, XD, RD, ND, DO'R, and CD: validation. SR and CD: resources. SR, RD, and ND: data curation. SR, HA, SB, JP, GS, JR, XD, RD, ND, DO'R, and CD: writing—review and editing. CD: supervision and funding acquisition.

### Conflict of Interest

The authors declare that the research was conducted in the absence of any commercial or financial relationships that could be construed as a potential conflict of interest.
