# Supplementary material for: Multivariate Analysis of Preoperative Magnetic Resonance Imaging Reveals Transcriptomic Classification of de novo Glioblastoma Patients
Source: Front Comput Neurosci. 2019 Dec 12;13:81. doi: 10.3389/fncom.2019.00081 (PMC6923885; doi:10.3389/fncom.2019.00081)
Supplement: Supplementary file 1 [file Data_Sheet_1.docx]

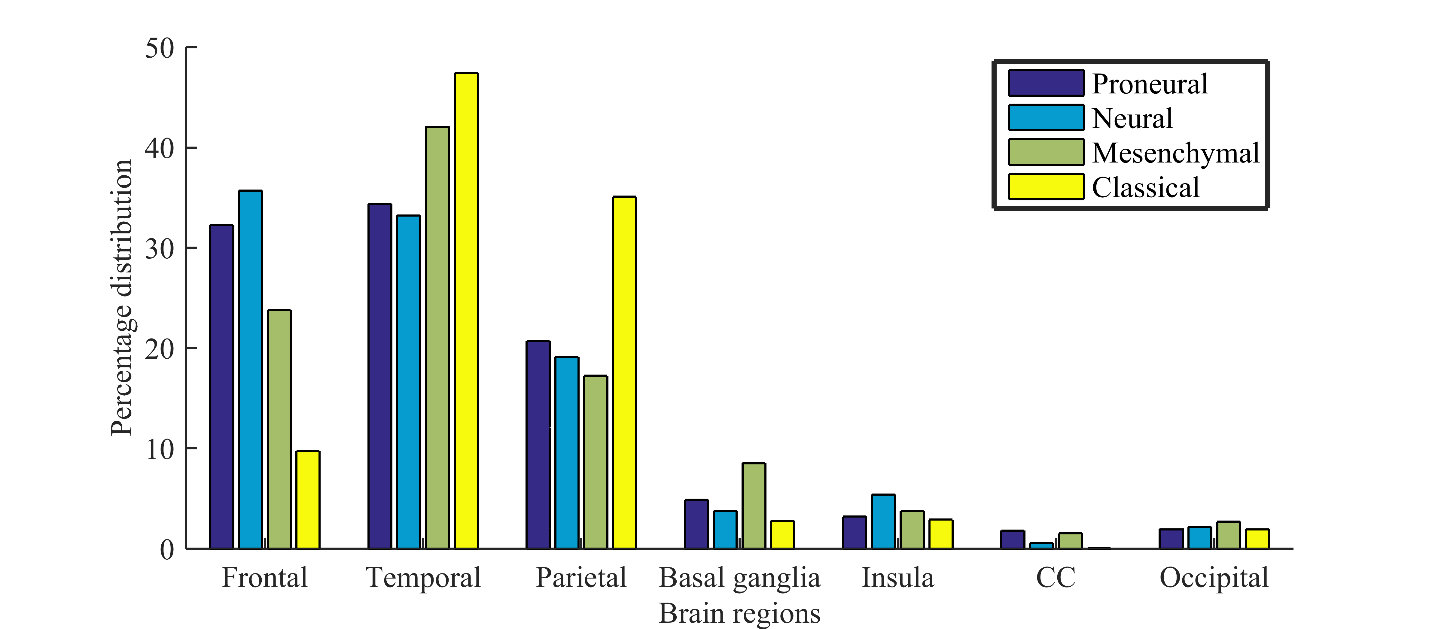


(A)


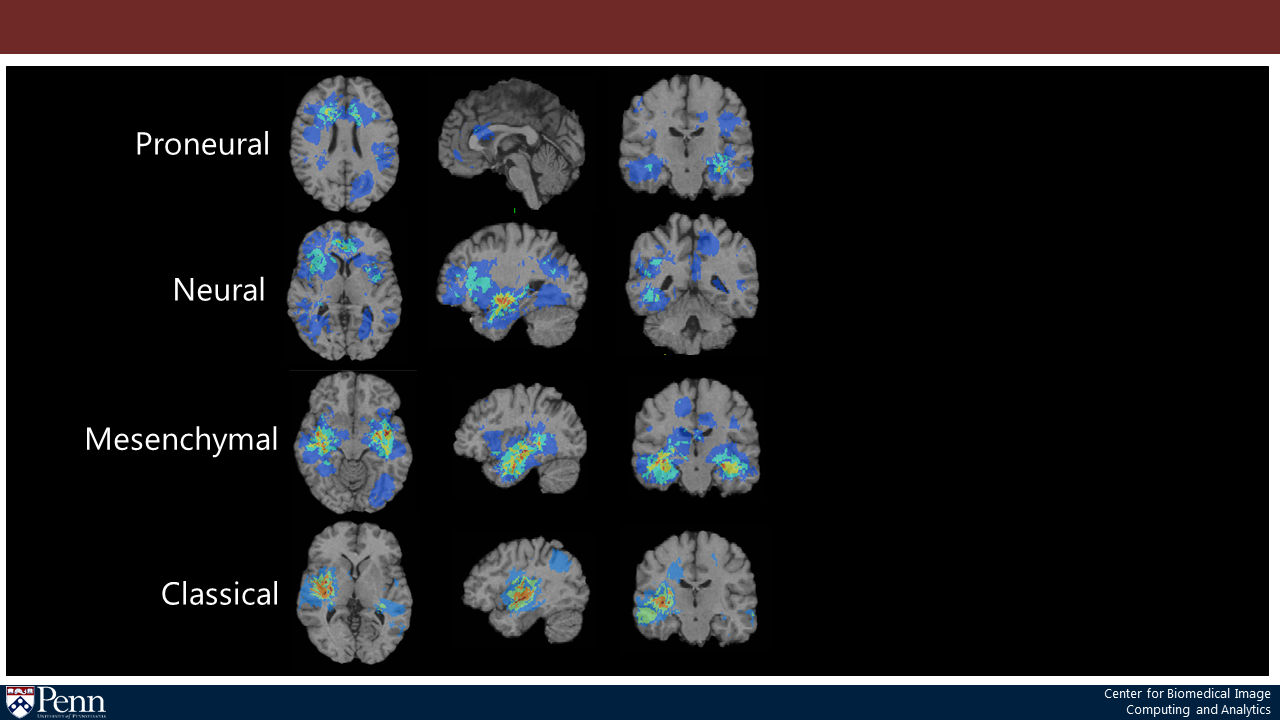


(B)

Figure S1: Spatial distribution of glioblastomas according to their molecular subtype status. (A) Percentage distribution of tumors in each brain region, and (B) Spatial distribution maps. The red and blue colors, respectively, show higher and lower frequencies.
